# Human Cytomegalovirus Nuclear Egress Complex Subunit, UL53, Associates with Capsids and Myosin Va, but Is Not Important for Capsid Localization towards the Nuclear Periphery

**DOI:** 10.3390/v14030479

**Published:** 2022-02-26

**Authors:** Adrian R. Wilkie, Mayuri Sharma, Margaret Coughlin, Jean M. Pesola, Maria Ericsson, Jessica L. Lawler, Rosio Fernandez, Donald M. Coen

**Affiliations:** 1 Department of Biological Chemistry and Molecular Pharmacology, Blavatnik Institute, Harvard Medical School, Boston, MA 02115, USA; adrian.r.wilkie@gmail.com (A.R.W.); mmayuri@gmail.com (M.S.); jean_pesola@hms.harvard.edu (J.M.P.); jlawler46@gmail.com (J.L.L.); rof36@pitt.edu (R.F.); 2 Committee on Virology, Harvard University, Cambridge, MA 02138, USA; 3 Department of Systems Biology, Blavatnik Institute, Harvard Medical School, Boston, MA 02115, USA; margaret_coughlin@hms.harvard.edu; 4 Electron Microscopy Core Facility, Department of Cell Biology, Blavatnik Institute, Harvard Medical School, Boston, MA 02115, USA; maria_ericsson@hms.harvard.edu

**Keywords:** capsid migration, human cytomegalovirus, UL53, UL50, myosin Va, major capsid protein, mass spectrometry, virus genetics, complementing cells, null mutants

## Abstract

After herpesviruses encapsidate their genomes in replication compartments (RCs) within the nuclear interior, capsids migrate to the inner nuclear membrane (INM) for nuclear egress. For human cytomegalovirus (HCMV), capsid migration depends at least in part on nuclear myosin Va. It has been reported for certain herpesviruses that the nucleoplasmic subunit of the viral nuclear egress complex (NEC) is important for this migration. To address whether this is true for HCMV, we used mass spectrometry and multiple other methods to investigate associations among the HCMV NEC nucleoplasmic subunit, UL53, myosin Va, major capsid protein, and/or capsids. We also generated complementing cells to derive and test HCMV mutants null for UL53 or the INM NEC subunit, UL50, for their importance for these associations and, using electron microscopy, for intranuclear distribution of capsids. We found modest associations among the proteins tested, which were enhanced in the absence of UL50. However, we found no role for UL53 in the interactions of myosin Va with capsids or the percentage of capsids outside RC-like inclusions in the nucleus. Thus, UL53 associates somewhat with myosin Va and capsids, but, contrary to reports regarding its homologs in other herpesviruses, is not important for migration of capsids towards the INM.

## 1. Introduction

Newly assembled herpesvirus capsids translocate from the nucleus to the cytoplasm in a complicated process known as nuclear egress. Nuclear egress includes four distinct steps: (1) migration of capsids from the nuclear interior, where viral genome replication and encapsidation occur within discrete replication compartments (RCs), to the nuclear rim; (2) disruption of the nuclear lamina providing access to the inner nuclear membrane (INM): (3) budding of capsids through the INM (primary envelopment); and (4) de-envelopment at the outer nuclear membrane (reviewed in [1,2,3,4]). 

Step 1 of this process—migration from the nuclear interior to the nuclear rim—is poorly understood. In one model, as initially proposed for an alphaherpesvirus, herpes simplex virus 1 (HSV-1) [5] capsids move from the nuclear interior towards the nuclear rim by actomyosin-directed transport. For a betaherpesvirus, human cytomegalovirus (HCMV), this model is supported for at least some intranuclear capsid migration by data showing that the virus induces the formation of actin filaments in the nucleus that associate with capsids and myosin Va, that treatment with an actin-depolymerizing drug disrupts these filaments and impairs migration to the nuclear rim and nuclear egress, and that siRNA and a nuclear localized dominant negative mutant that antagonize myosin Va also impair these processes [6,7]. However, how the capsid would interact with the actomyosin machinery to be transported from the nuclear interior to the nuclear rim remains unclear.

Another poorly understood aspect of nuclear egress is the relationship between this first step of nuclear egress and the viral nuclear egress complex (NEC). The NEC consists of two virus-encoded subunits, one that is anchored in the INM, and the other that binds to its nucleoplasmic face. For HCMV, the INM-anchored subunit is UL50 and the nucleoplasmic subunit is UL53. Both subunits are essential for viral replication and nuclear egress [8,9,10]. The HCMV NEC recruits the viral protein kinase, UL97, to the nuclear rim for phosphorylation and thus disruption of the nuclear lamina [10,11]. Based on work with other herpesvirus NECs, the HCMV NEC also likely orchestrates capsid budding during primary envelopment (reviewed in [1,2,3,4]). However, there is evidence that nucleoplasmic subunits of certain herpesvirus NECs participate in processes upstream of events at the nuclear rim, such as DNA packaging, and capsid migration to the nuclear periphery [12,13,14,15,16]. Moreover, homologs of UL53, such as the HSV-1 and pseudorabies virus nucleoplasmic NEC subunit, UL31, have been found to associate with intranuclear capsid proteins [16,17,18], and recently, some evidence that HCMV UL53 associates with intranuclear capsids was provided [19]. We were intrigued by these reports, as they raised the possibility that HCMV UL53 might interact with both capsids and the actomyosin machinery to mediate migration of HCMV capsids to the nuclear rim. 

This study began with a proteomics investigation to identify candidate proteins that interact with UL53. This identified possible associations of UL53 with myosin Va and with capsid proteins, and we found several lines of evidence for modest associations of these components that were consistent with the hypothesis that UL53 might serve as a bridge between the actomyosin machinery and capsids during their migration to the nuclear rim. We then generated complementing cell lines to allow us to derive stocks of viral mutants with sufficiently high titers to allow us to test whether UL53 was required for associations of myosin Va with capsids, and for capsid migration towards the nuclear rim. 

## 2. Materials and Methods

### 2.1. Cells and Viruses

Human foreskin fibroblasts (HFF) cells (ATCC, CRL-1684) and human embryonic kidney (293T) cells (ATCC (Manassas, VA, USA), CRL-11268) were propagated in DMEM containing 10% fetal bovine serum (FBS). The HCMV laboratory strain AD169 was used in all experiments. AD169-RV encoding a FLAG-tagged version of UL53 (53-F), and bacterial artificial chromosomes (BACs) encoding this virus and UL53-null (53N), UL50-null (50N), and UL53-null rescue-derivative (53NR) viruses have been described previously [10]. The production of HFFs expressing UL50 or UL53 and infectious 50N, 53N, and 53NR viruses is described below. Viruses were propagated and titrated as described previously [10,20].

### 2.2. Mass-Spectrometry

Immunoprecipitation (IP) of UL53-FLAG from nuclear lysates of infected cells for mass spectrometry was carried out as described previously [21]. Briefly, HFFs were infected with 53-F or WT HCMV (MOI 3) and cells were harvested at 72 h post-infection (hpi). Nuclear fractions were then isolated and subjected to α-FLAG IP. For mass spectrometry, eluates in Laemmli buffer were run on a 4–20% SDS-polyacrylamide gel. Extracted bands were submitted to the Taplin Mass-Spectrometry Facility (Harvard Medical School, Boston, MA, USA) for liquid chromatography-tandem mass-spectrometry (LC-MS/MS) analysis.

### 2.3. Immunoprecipitation

For immunoprecipitation (IP) of transfected cells, 5 × 10^6^ 293T cells/plate were seeded in 100 mm plates. Cells were then transfected with a pcDNA vector encoding a FLAG-tagged version of UL53 (UL53-FLAG) [22] and either a pcDNA-based plasmid encoding an HA-tagged version of UL50 (UL50-HA) [22] a lentiviral vector encoding green fluorescent protein with a nuclear localization sequence (GFP-NLS, [7]), or a lentiviral vector encoding the long tail of myosin Va fused to GFP-NLS (LT-GFP-NLS, [7]) (total of 10 μg DNA/plate; 1 μg/mL doxycycline was added to induce expression from the lentiviral vectors). At 48 h post-transfection, the cell monolayers were washed with Dulbecco’s phosphate-buffered saline (DPBS) and whole cell lysates were harvested in EBC buffer (50 mM Tris (pH 8.0), 150 mM NaCl, 0.5% NP-40) containing one Complete EDTA-free protease inhibitor tablet (Roche, Indianapolis, IN, USA) per 50 mL. For IP, 25 μL of EZ-View α-FLAG M2 affinity gel (Sigma, St. Louis, MO, USA) was added to lysates and rotated overnight at 4 °C. Resin was centrifuged and washed 4 times in 750 μL ice-cold EBC buffer by rotating for 20 min at 4 °C between washes. After the final spin, the resin pellet was mixed with 25 μL of EBC buffer, and protein was eluted from resin by incubation with 50 μL of 2x Laemmli buffer at 95 °C for 5 min and analyzed by Western blot as described below.

For IP of UL53-FLAG from infected cells, 2.5 × 10^6^ HFFs were infected with either 53-F or WT HCMV (MOI 1). At 72 hpi, cells were harvested, and nuclei were isolated and lysed using a Nuclear Complex Co-IP Kit (Active Motif, Carlsbad, CA, USA). The nuclear lysate was mixed with 1 mL EBC buffer containing one Complete EDTA-free protease inhibitor tablet (Roche) per 50 mL and precleared with 100 μL of mouse IgG-agarose (Sigma) by rotating at 4 °C for 5 h. For IP, 40 μL of EZ-View α-FLAG M2 affinity gel (Sigma) was added to precleared lysate and rotated overnight at 4 °C. Resin was centrifuged and washed 4 times in 750 μL ice-cold EBC buffer by rotating for 20 min at 4 °C between washes. After the final spin, the resin pellet was mixed with 40 μL of EBC buffer, and protein was eluted from resin by incubation with 80 μL of 2x Laemmli buffer at 95 °C for 5 min and analyzed by Western blot as described below.

### 2.4. Western Blotting

For Western blotting of IPs, lysates and eluates in Laemmli buffer were separated on a 4–20% SDS-polyacrylamide gel (Bio-Rad, Hercules, CA, USA). Proteins were then transferred onto a PVDF membrane, blocked with 5% milk in DPBS-T (DPBS with 0.5% Tween-20), and probed with primary antibodies (see below for sources and dilutions) overnight at 4 °C with rocking. Membranes were washed 3x with DPBS-T for 10 min at room temperature (RT) with rocking. Membranes were then incubated with TrueBlot secondary antibodies conjugated to horseradish peroxidase (HRP) (Rockland, Limerick, PA, USA) at 1:1000 for 1 h at RT with rocking, followed by washing. Finally, Pierce chemiluminescence solution (ThermoFisher, Waltham, MA, USA) was added to membranes and signal was detected with film.

For all other Western blotting, cells were harvested by washing with DPBS followed by the addition of 2x Laemmli buffer with protease inhibitors (ThermoFisher) directly to the monolayer. Lysates were scraped off the plate, boiled at 95 °C for 5 min, and processed as described above.

Primary antibody dilutions were as follows: rabbit α-myosin Va (Cell Signaling Technology, Danvers, MA, USA, #3402), 1:1000; mouse α-β actin (Sigma A5441), 1:5000; mouse α-FLAG M2 (Sigma F1804), 1:100; rabbit α-UL53 [10], 1:500; rabbit α-UL50 [10], 1:500; rabbit α-PCNA (Abcam, Cambridge, UK, ab18197), 1:700; mouse α-MCP (a kind gift from William Britt, University of Alabama, Birmingham, AL, USA), 1:1000; rabbit α-GFP (ThermoFisher A11122), 1:1000.

### 2.5. Immunofluorescence Analysis

For immunofluorescence, 1 × 10^5^ HFFs/well were seeded on glass coverslips in a 24-well plate followed by either mock infection or infection with WT, 53-F, 50N/53F HCMV (MOI 1) as indicated in the text. At the time-points indicated, cells were fixed at RT in 3.7% formaldehyde/DPBS. Cells were then permeabilized at RT in 0.1% Triton X-100/DPBS, washed 3x with DPBS, and blocked overnight in a mixture of 1% bovine serum albumin (Sigma) and 5% human serum (Sigma) in DPBS. The following antibodies and dilutions were used for primary staining: rabbit α-myosin Va (Abcam, ab11094), 1:50; mouse α-FLAG M2 (Sigma F1804), 1:500; mouse α-MCP (a kind gift from William Britt, University of Alabama, Birmingham, AL, USA), 1:250. Antibodies were diluted in a mixture of 1% BSA/5% human serum in DPBS and added to coverslips for 1 h at RT with rocking. Primary antibodies were removed and coverslips washed 3x with DPBS for 5 min with rocking at RT. The staining procedure was repeated with the appropriate fluorescently labeled Alexa-fluor secondary antibodies (ThermoFisher), and DAPI was applied in the last 10 min of the secondary antibody incubation. After the final washes, coverslips were mounted on glass slides using ProLong Anti-fade (ThermoFisher). Imaging was carried out at the Nikon Imaging Center (NIC) at Harvard Medical School using a Nikon Ti spinning-disk confocal laser microscope. Postacquisition image analysis was conducted using Metamorph and ImageJ software packages.

### 2.6. Immunoelectron Microscopy

Here, 2 × 10^5^ HFFs/well were seeded in 12-well plates and infected with either WT or 53-F HCMV (MOI 1). At 72 hpi, cells were washed with DPBS, trypsinized, and harvested. The cell suspension was layered on top of a cushion of 4% paraformaldehyde and 0.1% glutaraldehyde in DPBS and pelleted for 3 min at 3000 rpm. The supernatant was carefully removed and fresh 4% paraformaldehyde and 0.1% glutaraldehyde were added. After fixation at RT for 2 h, the fixative was replaced with DPBS. Prior to freezing in liquid nitrogen the cell pellets were infiltrated with 2.3 M sucrose in DPBS (containing 0.2 M glycine to quench free aldehyde groups) for 15 min. Frozen samples were sectioned at −120 °C, the sections were transferred to formvar-carbon coated copper grids. Grids were floated on DPBS or stored on 2% gelatin dishes at 4 °C until immunogold labeling. The gold labeling was carried out at RT on a piece of parafilm. Antibodies and protein A gold were diluted in 1% BSA in PBS. The diluted primary antibody solution (α-myosin Va 1:10) was centrifuged 1 min at 14,000 rpm prior to labeling to avoid possible aggregates. Grids were floated on drops of 1% BSA for 10 min to block for unspecific labeling, transferred to 5 µL drops of primary antibody and incubated for 30 min. The grids were then washed in 4 drops of DPBS for a total of 15 min, transferred to 5 µL drops of 10 nm Protein A gold for 20 min, washed in 4 drops of DPBS for 15 min and 6 drops of double-distilled water. Contrasting/embedding of the labeled grids was carried out on ice in 0.3% uranyl acetate in 2% methyl cellulose for 10 min. Grids were picked up with metal loops (diameter slightly larger than the grid) and the excess liquid was removed by streaking on filter paper, leaving a thin coat of methylcellulose.

The grids were examined on a JEOL 1200EX electron microscope and images were recorded with an AMT 2k CCD camera. Labeled and unlabeled capsids were counted, and the percentage of capsids associated with at least one gold particle was calculated for each condition, and analyzed by Fisher’s exact test using GraphPad Prism Version 7 software, GraphPad Software, San Diego, CA, USA.

### 2.7. Generation of Infectious 53N and 50N Viruses

To generate HFFs stably expressing either UL53 or UL50, each viral gene was amplified from WT HCMV BAC DNA with the following primers: UL53, forward: 5′-TAAGCAGCGGCCGCATGTCTAGCGTGAGCGGC GTGCGCA-3′; UL53, reverse: 5′-TGCTTAGGATCCTCAAGGCGCACGAATGCTGTTGAGAAACAGCGG-3′; UL50, forward: 5′-TAAGCAGCGGCCGCATGGAGATGAACAAGG TTCTCCATC-3′; UL50, reverse: 5′-TGCTTAGGATCCTCAGTCGCGGTGTGCGGAGCGTGTCGGA-3′. Each PCR product was digested with Not1 and BamH1 restriction enzymes and cloned into the pLVX-eF1αlentiviral vector (generous gift from the late Gregory Pari, University of Nevada, Reno, NV, USA). Lentiviruses were produced following transfection of 293T with these pLVX-eIFα-based plasmids and used to transduce HFFs as described previously [7]. The UL53 expressing cell lines were then electroporated with WT, 53N, or 53NR BACS, and the UL50 expressing cell line was electroporated with WT or 50N HCMV BAC DNA. After several weeks, viral supernatant was harvested and used for experiments as described in the text.

### 2.8. Transmission Electron Microscopy

Transmission electron microscopy (TEM) was utilized to assess subnuclear capsid distribution by counting capsids in the nuclei either inside or outside of RC-like inclusions under the conditions described in the text using a TecnaiG² Spirit BioTWIN electron microscope equipped with an AMT 2k CCD camera. Processing for image acquisition was performed as described previously [23]. Intranuclear capsid distributions were assessed by counting capsids within or outside of electron dense inclusions in the interior of the nucleoplasm that have been considered to be RCs (e.g., [15]) in representative sections of whole nuclei [6]. We term these RC-like inclusions. Data analyses for capsid distributions were performed as described previously [23], using ordinary one-way ANOVA corrected for multiple comparisons using the Holm–Sidak test, while analyses of capsid counts were performed using the Kruskal–Wallis test and Dunn’s tests for multiple comparisons. All statistical analyses used GraphPad Prism version 7 (capsid distributions) and version 9.3.1 for Mac (capsid counts).

### 2.9. Correlative Light Electron Microscopy

Correlative light electron microscopy (CLEM) was conducted as described previously [10]. Briefly, HFFs were electroporated with either 53N or 53NR BAC DNA. The following day, cells were seeded onto gridded glass bottom dishes, and on day 7 or 8 postelectroporation they were fixed, imaged with fluorescence and phase microscopy to visualize the electroporated cells and the grid, and processed for EM. GFP-positive cells were identified by their grid coordinates, excised, and remounted for serial sectioning. Imaging was carried out using a TecnaiG^2^ Spirit BioTWIN microscope. Representative whole-cell sections from three GFP-positive cells containing capsids were analyzed for each condition. Fisher’s exact test was applied to data using GraphPad Prism software version 7.

## 3. Results

### 3.1. Mass Spectrometry Identifies Potential UL53 Binding Partners

**Figure 1:**
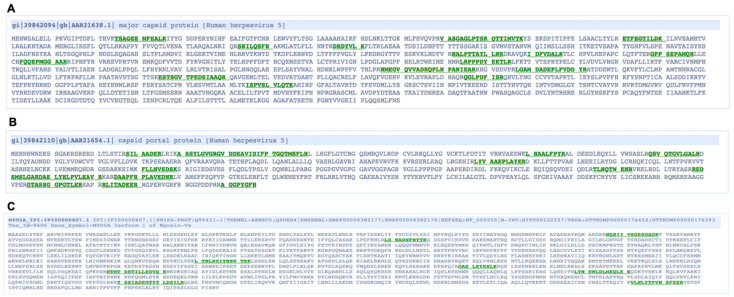
HFFs were infected with 53-F (MOI 3), nuclear lysates were harvested at 72 hpi, and subjected to α-FLAG IP. Proteins were resolved on an SDS-polyacrylamide gel and extracted bands were sent for LC-MS/MS analysis. Sequences of major capsid protein (**A**), capsid portal protein (**B**), and myosin Va (**C**) are shown with positions of unique peptides detected in the LC-MS/MS analysis highlighted in green.

To identify possible viral and cellular binding partners of UL53 in the nuclei of infected cells, human foreskin fibroblasts (HFFs) were infected with HCMV expressing a FLAG-tagged version of UL53 (53-F; multiplicity of infection (MOI) 3), or as a control, wild type (WT) HCMV, and, at 72 hpi, nuclear lysates were prepared and immunoprecipitated using α-FLAG antibody conjugated resin [21]. Immunoprecipitates were then subjected to liquid chromatography-tandem mass-spectrometry (LC-MS/MS) analysis, which uncovered peptides derived from numerous viral and cellular proteins in nuclear lysates from 53F-infected but not WT-infected cells. Of particular interest for this study were major capsid protein (MCP; 20 peptides; 13% coverage; peptides shown in Figure 1A) the primary protein constituent of capsids, and the capsid portal protein (UL104; 15 peptides; 23% coverage; peptides shown in Figure 1B). Also of interest for this study was the cellular protein myosin Va (MyoVa; 8 peptides; 6% coverage; peptides shown in Figure 1C), an F-actin-based host motor protein that we have previously shown is important for nuclear egress at the stage of localization towards the nuclear rim [7]. A list of viral proteins identified in this study is presented in Table S1 in Supplementary Materials. A similar list of cellular proteins will be reported separately.

### 3.2. Co-Immunoprecipitation of UL53, Myosin Va, and Major Capsid Protein

**Figure 2:**
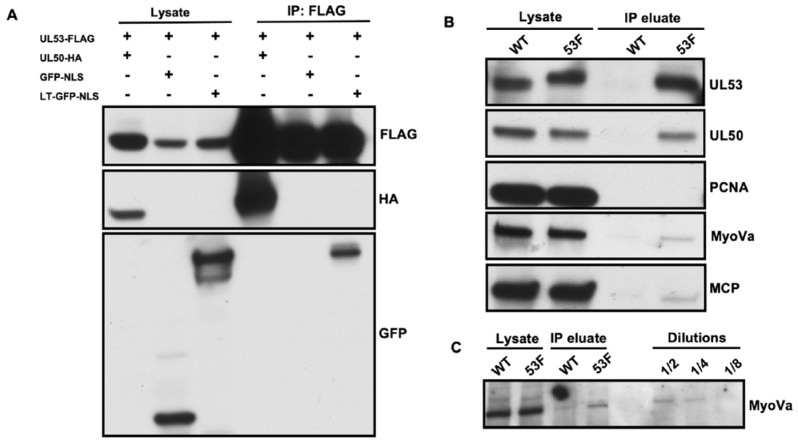
(**A**) 293T cells were co-transfected with a plasmid expressing UL53-FLAG together with a plasmid encoding the myosin Va long tail (LT) fused to GFP with an NLS (LT-GFP-NLS), GFP-NLS (negative control), or UL50-HA (positive control). UL53-FLAG was immunoprecipitated from whole cell lysates with α-FLAG IP and immunoprecipitates were analyzed by Western blot using the antibodies indicated at the right of each panel. (**B**) HFFs were infected with 53-F or WT HCMV (MOI 1). At 72 hpi, nuclear lysates were prepared, subjected to α-FLAG IP, and lysates and eluates were analyzed by Western blot using antibodies against the proteins indicated to the right of each panel. (**C**) The 53-F immunoprecipitates from above were diluted as indicated and analyzed by Western blot for myosin Va (MyoVa).

Given the reports that UL53 homologs of herpes simplex virus-1 (HSV-1), Epstein–Barr virus (EBV), and mouse CMV participate in the process of capsid migration [13,15,16], and our previous findings that myosin Va interacts with capsids and is important for such migration [7], we were intrigued by this finding. We therefore investigated whether associations of UL53 with myosin Va and MCP detected by MS (which could be direct or indirect) could also be detected using other assays. We began by asking whether UL53 can associate with the long tail (LT) region of myosin Va that contains the cargo-binding globular tail domain (GTD). To that end, we co-transfected 293T cells with a plasmid encoding UL53-FLAG together with a plasmid expressing LT fused to green fluorescent protein (GFP) with a nuclear localization signal (LT-GFP-NLS), GFP-NLS as a negative control, or UL50-HA as a positive control. UL53-FLAG was then immunoprecipitated from whole cell lysates with α-FLAG resin and immunoprecipitates were analyzed by Western blot using anti-FLAG antibodies to detect UL53, anti-HA antibodies to detect UL50, and anti-GFP antibodies to detect LT-GFP-NLS and GFP-NLS. We readily detected UL50-HA and LT-GFP-NLS in UL53-FLAG immunoprecipitates, but did not detect GFP-NLS, indicating that UL53 can associate directly or indirectly with myosin Va (Figure 2A).

We then investigated whether we could detect associations between UL53 and MCP and/or endogenous myosin Va in HCMV-infected cells. HFFs were infected with either 53-F HCMV, or as a negative control to detect nonspecific associations, WT HCMV (MOI 1). Nuclear lysates were prepared at 72 hpi and subjected to α-FLAG immunoprecipitation (IP), and lysates and eluates were analyzed by Western blot using antibodies against MCP, myosin Va, UL50 (as a positive control) and PCNA (as a negative control (Figure 2B). We found that UL53 immunoprecipitated from lysates of 53-F, but not WT infected cells, as expected. Also as expected, UL50 co-immunoprecipitated with UL53, while the host protein, PCNA, which is not known to bind UL53, did not. We detected MCP and myosin Va in Western blots of immunoprecipitates from 53-F infected nuclear lysates. We could also detect these proteins in immunoprecipitates from WT infected nuclear lysates, indicating background, nonspecific associations, but at lower levels (for myosin Va, ~3 to 4-fold lower based on a dilution series; Figure 2C), indicating that the majority of the MCP and myosin Va found in FLAG IP from nuclear lysates of 53-F infected cells arose from specific associations. These data confirm the MS results, suggesting modest associations, which could be direct or indirect, of UL53 with myosin Va and MCP in nuclear lysates.

### 3.3. A Population of UL53 Colocalizes with Myosin Va and Capsids in the Nucleoplasm

**Figure 3:**
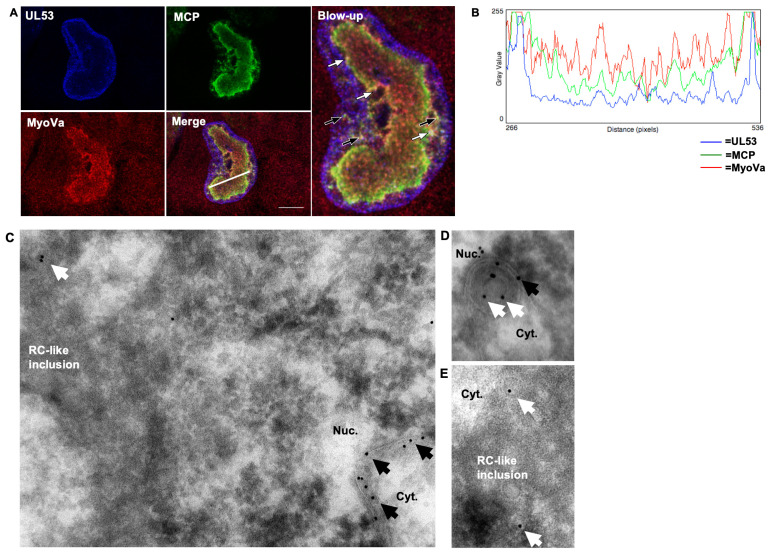
(**A**) HFFs were infected with 53-F HCMV (MOI 1) and fixed at 72 hpi. Cells were then stained with α-FLAG (blue), α-MCP (green), and α-myoVa (red) antibodies and imaged with spinning-disk confocal microscopy. Yellow color indicates colocalization between myosin Va (MyoVa) and MCP; purple color indicates colocalization between myosin Va and UL53; cyan color indicates colocalization between UL53 and MCP; white color and arrows indicate colocalization between UL53, MCP, and myosin Va (black arrows indicate examples of colocalization between the RC periphery and the nuclear rim; white arrows indicate examples of colocalization at the nuclear rim). Images are single optical sections. Scale bar is 10μm. (**B**) To measure colocalization between UL53, MCP, and myosin Va at the RC periphery, the fluorescence intensity of each channel was plotted across the indicated white line. (**C**–**E**) HFFs were infected with 53-F HCMV (MOI 1) and fixed for immunoEM at 72 hpi. The cells were further processed by primary staining with an α-FLAG antibody, followed by secondary staining with a 10nm protein A-gold secondary. Imaging was conducted using a transmission electron microscope. Black arrows indicate UL53 associated with the INM. White arrows indicate UL53 associated with capsids.

To study possible associations of UL53, MCP, and myosin Va by a different method, we examined the localization of UL53, MCP, and myosin Va in infected cells, initially using immunofluorescence assays (IFA). HFFs were infected with 53-F HCMV (MOI 1) and at 72 hpi cells were fixed, stained with α-myosin Va, α-MCP, and α-FLAG antibodies, and single optical sections were imaged with confocal microscopy (Figure 3A). As expected, most UL53 was found at the nuclear rim; however, a population was also present in the nucleoplasm. We have previously shown that myosin Va concentrates in replication compartments (RCs) [7], and the staining pattern observed in this experiment was consistent with that localization. MCP also concentrated at the periphery of these structures where it colocalized with myosin Va (yellow in merged image). We observed some colocalization of UL53 with myosin Va and MCP both at the periphery of RCs and between the RCs and nuclear rim, but not at the rim (white in the merged image). Colocalization of the three proteins at the periphery of RCs was verified by measuring the fluorescence intensity of each channel (Figure 3B).

We then utilized immunoelectron microscopy (immunoEM) to assess UL53 association with capsids at the ultrastructural level. We infected HFFs with 53-F HCMV (MOI 1), fixed cells at 72 hpi, and processed cells by staining with an α-FLAG antibody followed by 10 nm protein A-gold secondary staining. Consistent with our IFA findings, most UL53 could be found at the nuclear rim where it concentrated in areas where the INM appeared slightly infolded (Figure 3C). Furthermore, these infoldings occasionally contained capsids in the perinuclear space likely in the process of capsid budding (Figure 3D), consistent with a previous report [24]. We also observed a population of UL53 in the nucleoplasm that associated with capsids in RC-like inclusions, consistent with our IFA results (Figure 3C,E). Thus, these IFA and immunoEM data provide further evidence that UL53 modestly associates with capsids in the nucleoplasm, consistent with the modest associations seen in co-immunoprecipitation experiments.

### 3.4. Generation of UL53 and UL50 Null Mutants Using Complementing Cell Lines

We wondered how UL53 would localize relative to myosin Va and MCP in the absence of UL50, and we especially wished to test whether UL53 is necessary for capsid localization to the nuclear rim, and for myosin Va association with capsids. We attempted to use correlative light-electron microscopy (CLEM) of HFFs electroporated with bacterial artificial chromosomes (BACs) expressing GFP and containing *UL53*-null (53N) HCMV or a rescued derivative (53NR), fixing at 7 or 8 days postelectroporation (dpe), and imaging with phase and fluorescence microscopy. (We had previously used these BACs to study infection in the absence or presence of UL53 [10]). Based on the grid coordinates of GFP-positive cells, we then traced back their location using EM and imaged capsids in representative whole cell sections (Figure S1 in Supplementary Materials). We observed a lower percentage of capsids located within 2 µm of the nuclear rim in 53N versus 53NR electroporated cells, and taken at face value, this difference was statistically significant (Figure S1 in Supplementary Materials). However, we did not trust that this difference was meaningful as we only were able to observe a total of three cells per condition, and some electroporated cells contained many fewer capsids than others and did not display obvious RC-like inclusions (Figure S1), suggesting that infection was not at the same stage for all cells.

**Figure 4:**
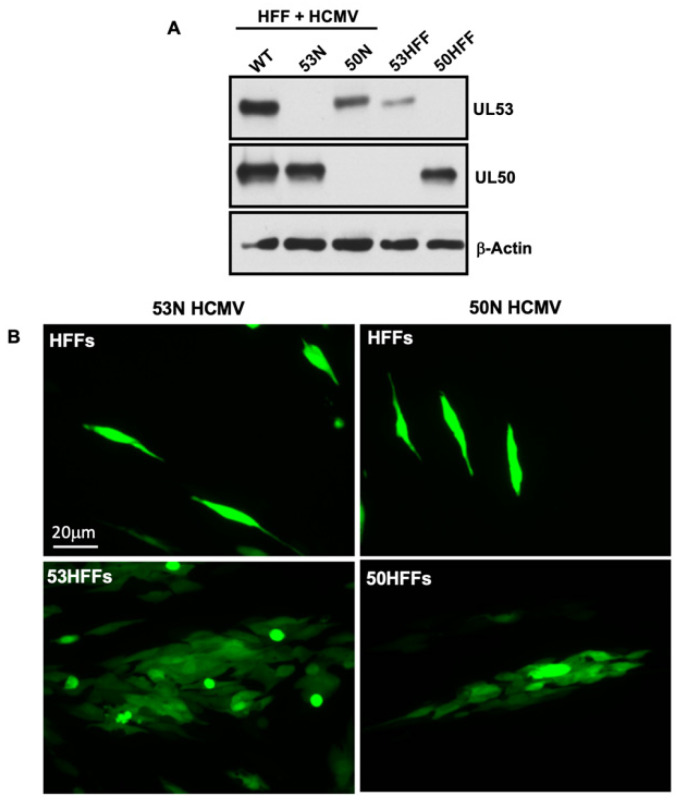
(**A**) HFFs were transduced with lentiviruses expressing UL53 or UL50 creating 53HFFs or 50HFFs, respectively. Expression of UL50 and UL53 in whole cell lysates of WT-infected cells (leftmost lane) was compared with that in uninfected 53HFF and 50HFF (rightmost two lanes), and with HFFs infected with 53N or 50N (second and third lanes from left, respectively) by Western blot, with β-actin as a loading control. Infected cells were harvested at 72 hpi. Cells and viruses used are indicated at the top of the image. Antibodies used are indicated to the right of the image. (**B**) Noncomplementing HFFs (top rows) and 53HFFs or 50HFFs (bottom rows) were electroporated with 53N or 50N GFP BAC DNA and imaged with widefield fluorescence microscopy to assess virus spread at 7 dpe.

Given these results and as CLEM is labor intensive, time consuming, and able to examine only a few cells per experiment, we instead generated infectious 50N HCMV that expresses either untagged (50N) or a FLAG-tagged UL53 (50N/53-F) and also *UL53*-null (53N) virus using complementing UL50-expressing (50HFFs) and UL53-expressing (53HFFs) cells, respectively. Previous attempts in our lab to generate complementing HFFs using a retroviral transduction system were unsuccessful; thus we opted to use the pLVX-eF1α lentiviral vector that had previously been used to complement HCMV *UL84* null virus growth in HFFs [25]. Following transduction and puromycin selection, we found that 50HFFs and 53HFFs expressed UL50 or UL53, respectively, using Western blot (Figure 4A). We then electroporated HFFs with either 53N or 50N BAC DNA and observed virus spread in complementing but not noncomplementing HFFs, as expected (Figure 4B). Infection of noncomplementing HFFs with infectious 53N or 50N HCMV confirmed that each virus did not detectably express UL53 or UL50, respectively (Figure 4A). After concentration by ultracentrifugation, we were able to generate relatively high titer stocks (~10^6^ PFU/mL) of the null mutant viruses using the complementing cells. A recent paper has described the generation of cells that express UL50 and complement a *UL50* null mutant [26].

### 3.5. Increased Association of UL53 with Major Capsid Protein and Myosin Va in the Absence of UL50

**Figure 5:**
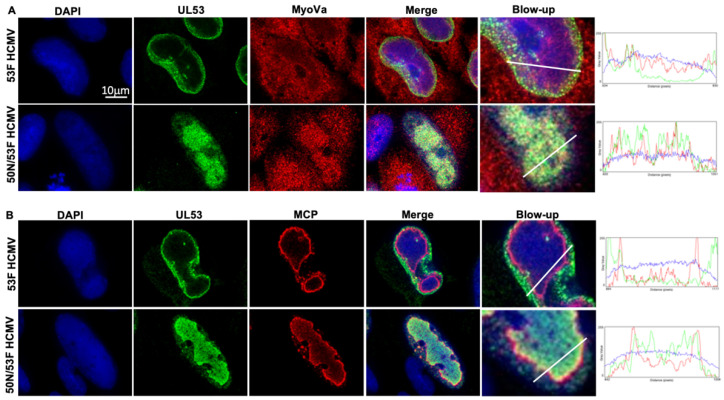
HFFs were infected with 53-F or 50N/53-F HCMV (MOI 1) and fixed at 72 hpi. Cells were then stained with DAPI (blue), α-FLAG (green), and in (**A**) α-myoVa (red) antibodies, or in (**B**) α-MCP (red) antibodies, and imaged with spinning-disk confocal microscopy. Yellow color indicates colocalization of UL53 with myoVa or MCP. Colocalization was measured by plotting fluorescence intensity of each channel across the indicated white lines. Images are single optical sections.

We next infected HFFs with 53-F or 50N/53-F HCMV (produced in 50HFFs) and fixed cells for confocal microscopy at 72 hpi. As both viruses also express GFP, there were not enough laser channels to image UL53, MCP, and myosin Va together in these samples. Therefore, we stained cells with DAPI, α-FLAG, and either α-myosin Va or α-MCP antibodies. As was seen in Figure 3, most UL53 was found at the nuclear rim during 53-F infection; however, a subpopulation was present in the nucleoplasm where it colocalized with myosin Va (Figure 5A) or MCP (Figure 5B), consistent with localization at the RC periphery and between the RC periphery and the nuclear rim. Notably, we found that in the absence of UL50 following infection with 50N/53F, essentially no UL53 was present at the nuclear rim and there was much more obvious colocalization of nucleoplasmic UL53 with myosin Va (Figure 5A) or MCP (Figure 5B), again consistent with all three proteins localizing at the RC periphery. Collectively, these data provide further evidence for associations of UL53 with myosin Va and MCP at the periphery of the RC.

### 3.6. UL53 Is Not Required for the Association between Capsids and Myosin Va

**Figure 6:**
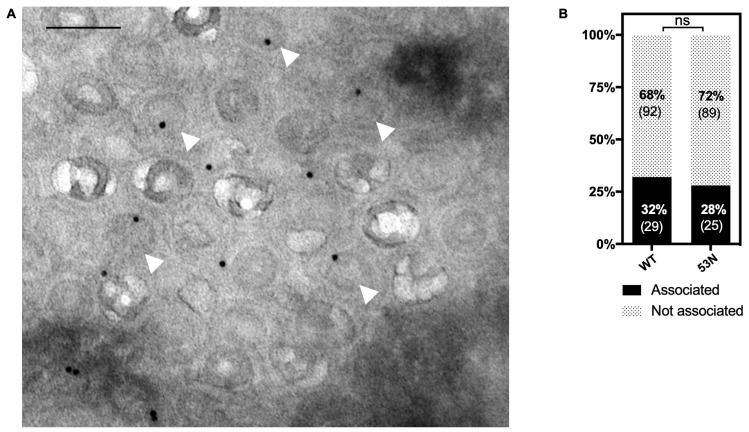
HFFs were infected with either WT or 53N HCMV (MOI 1) and fixed for immunoEM at 72 hpi. The cells were further processed by primary staining with α-myoVa followed by secondary staining with 10-nm protein A-gold. Imaging was conducted using a transmission electron microscope. (**A**) shows a representative image of the 53N condition. White arrowheads indicate capsids that are associated with myoVa. Scale bar is 100 nm. (**B**) The percentage of capsids associated with at least one gold particle was calculated for each condition (WT, *n* = 121; 53N, *n* = 144). The *p* value was calculated using Fisher’s exact test (ns = not significant, *p* = 0.76).

The association of UL53 with capsids (Figure 3C–E) and with myosin Va (Figure 2 and Figure 3A,B) evoked the hypothesis that this NEC subunit might serve as a bridge between capsids and the actomyosin system for intranuclear transport. We had previously shown that myosin Va interacts with capsids in HCMV-infected cells using immunoEM [7]. We therefore conducted immunoEM to determine whether UL53 is important for this association. HFFs were infected with WT or 53N HCMV (MOI 1), fixed at 72 hpi, and processed for immunoEM by staining with α-myosin Va antibodies followed by secondary staining using 10nm protein A-gold. We observed that myosin Va associated with a similar percentage (~30%) of nuclear capsids in both WT- and 53N-infected cells (Figure 6). We thus conclude that UL53 is not required for the association between capsids and myosin Va.

### 3.7. UL53 Is Not Important for Capsid Localization Away from RC-like Inclusions

**Figure 7:**
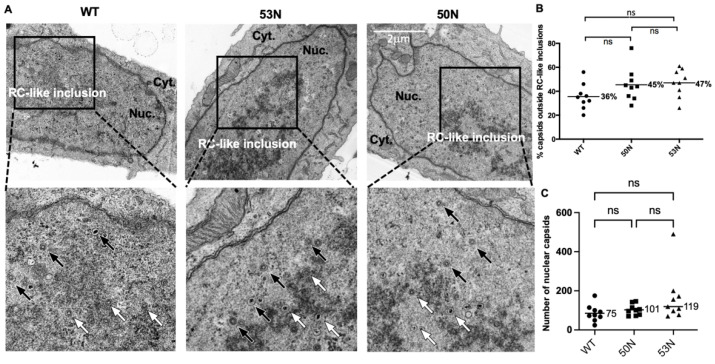
(**A**) HFFs were infected with WT, 50N, or 53N HCMV (MOI 1) and at 72 hpi cells were fixed and processed for EM. Black arrows point to capsids outside RC-like inclusions. White arrows point to capsids associated with RC-like inclusions (**B**) The percentage of capsids located outside RC-like inclusions was calculated in sections of 9 nuclei per condition and plotted. The horizontal bars indicate the mean percentage of capsids for each condition. *p*-values were calculated using ordinary one-way ANOVA corrected for multiple comparisons using the Holm–Sidak test (ns = not significant. WT vs. 50N, *p* = 0.18; WT vs. 53N, *p* = 0.16; 50N vs. 53N, *p* = 0.79). (**C**) The number of capsids found in the same sections analyzed in (**B**). The horizontal bars indicate the median numbers of capsids per section; the medians were calculated to obviate misleading skewing of the results by an outlying data point. Data were analyzed using a Kruskal–Wallis test with Dunn’s tests to provide *p*-values corrected for multiple comparisons (ns = not significant). WT vs. 50N, *p* = 0.8346; WT vs. 53N, *p* = 0.0806; 50N vs. 53N, *p* = 0.7768).

Our finding that UL53 was not important for myosin Va association with capsids led us to investigate whether UL53 facilitates HCMV capsid migration to the nuclear rim, as has been reported for UL53 homologs of some other herpesviruses [13,15,16] and suggested for HCMV UL53 [19]. To address this question, we infected HFFs with stocks of WT, 53N, or 50N HCMV (MOI 1). Cells were fixed and processed for EM at 72 hpi. RC-like inclusions were apparent in the nuclei of all cells analyzed (examples in Figure 7A), which allowed us to evaluate capsid distribution relative to the inclusions as we had done previously [7]. We observed no decrease and, if anything, a slight increase in the percentage of capsids located outside RC-like inclusions in cells infected with 53N or 50N virus compared to WT, although this difference was not significant (Figure 7B). The mutant-infected cells contained roughly similar numbers of nuclear capsids as WT-infected cells (Figure 7C), as we have observed with other mutants defective for nuclear egress (e.g., [23]). Thus, UL53 is not important for capsid localization away from RC-like inclusions.

## 4. Discussion

How nascent herpesvirus capsids migrate from RCs in the nuclear interior to the periphery during nuclear egress is poorly understood. It is particularly unclear whether the NEC or its subunits, which are crucial for later steps of nuclear egress, play a role in this migration. We performed a mass spectrometry study to look for proteins that associate directly or indirectly with the nucleoplasmic subunit of the HCMV NEC, UL53, and identified myosin Va and capsid proteins as candidate UL53-interacting proteins. Follow-up studies provided evidence to validate these associations, although they were rather modest. Our results together with results reported previously in various herpesvirus systems [5,6,7,12,13,14,15,16,19], led to the hypothesis that UL53 might serve as a bridge between capsids and nuclear actomyosin machinery. To test this role for UL53 and, more generally, a role in migration from the nuclear interior to the nuclear rim, we generated stocks of *UL50*- and *UL53*-null viruses using newly derived complementing cells. Experiments using these mutant viruses led to the conclusion that UL53 is neither important for associations between capsids and myosin Va nor for capsid localization away from RC-like inclusions in the nuclear interior. We discuss each of these findings below.

Our mass spectrometry, co-IP, IFA, and immunoEM results suggested associations among UL53, myosin Va, and major capsid proteins or assembled capsids in infected nuclei. By IFA and immunoEM, during WT infection, myosin Va and MCP or capsid associations were found largely within RCs or RC-like inclusions in the nuclear interior, while most UL53 could be found at the nuclear rim (Ref.[7] and this study). However, even during WT infection, a subpopulation of UL53 was also evident in the nuclear interior where it colocalized with myosin Va and MCP (as shown by IFA) or capsids (as shown by immunoEM—the IFA studies do not distinguish between capsids and unassembled MCP or partially assembled particles). This is consistent with reports that the HSV-1 homolog of UL53 (HSV-1 UL31), co-localizes with MCP hexons and associates with the portal vertex of capsids in the nucleoplasm [16,17]. The results are also consistent with a report that HCMV UL53 could be found in co-IP using antibodies against smallest capsid protein, and on intranuclear capsids by immunoEM [19]. However, the capsids shown in that report were close to the nuclear envelope rather than in RC-like inclusions. Regardless, the relatively small amount of nucleoplasmic UL53 involved in associations with myosin Va and capsids likely reflects the small proportion that is not bound to UL50 at the INM under steady state conditions. Consistent with that surmise, UL53 primarily localizes to RCs during 50N infection, where we detected more obvious colocalization of UL53 with myosin Va or MCP.

Thus, we speculate that, ordinarily, some UL53 and myosin Va associate initially with capsids in RCs, and that subsequent movement to the nuclear periphery and primary envelopment occur relatively quickly and are thus not easily detected in fixed cells. For interactions between UL53 and capsids, there is considerable evidence for association of UL53 homologs of other viruses with various capsid proteins, particularly HSV-1 UL31 with HSV-1 UL25, or inner tegument proteins [17,18,27,28,29,30]. A recent study made the exciting finding that in HSV-1, UL25, interacts with the NEC and promotes formation of pentagonal arrays, which are posited to anchor capsids to the NEC and promote NEC curvature [30]. The HCMV homolog of HSV-1 UL25, UL77, was not one of the proteins detected in our MS study, although we did detect MCP, the portal protein UL104, and several other components of intranuclear capsids including the terminase subunits, UL56 and UL89, and the inner tegument protein, UL32 (pp150), as well as TRS1, which has been reported to abet capsid assembly (Table S1 in Supplementary Materials). Our failure to detect UL77 may reflect limitations of the mass spectrometric approach, our use of soluble nuclear lysates, or differences in the HCMV and HSV-1 systems.

In contrast with the literature on associations of UL53 homologs with capsids and/or capsid proteins, there is little evidence regarding how myosin Va associates with these and with UL53. Such associations could be direct or indirect. Given these associations and previous studies indicating a role for UL53 homologs and myosin Va in intranuclear distribution of capsids [7,15,16] the hypothesis that UL53 might serve as an adaptor protein, bridging capsids and myosin Va, was attractive. However, deletion of UL53 did not discernibly affect the association between capsids and myosin Va. Thus, the details of UL53 interactions with myosin Va and nucleoplasmic capsids remain unclear.

Our finding that a population of UL53 localizes to the nucleoplasm was consistent with reports that UL53 homologs participate in events upstream of primary envelopment. For example, roles for UL53 homologs in viral DNA packaging (or stabilization of filled capsids) have been described for α-, β-, and γ-herpesviruses [12,13,14,15]. Furthermore, UL53 homologs have been suggested to facilitate capsid migration to the nuclear periphery. In one paper, it was stated in the Discussion that in cells replicating Epstein–Barr virus genomes lacking its *UL53* homolog, capsids were homogeneously distributed throughout the nucleus rather than being mostly aligned along the nuclear membrane [13]. In a second paper, in one of the figures, capsids derived from MCMV expressing a dominant-negative version of its UL53 homolog, M53, clustered in the nuclear interior away from the nuclear rim [15]. These authors went on to suggest that impaired viral DNA packaging observed with this mutant (and with another dominant-negative *M53* mutant [14]) causes capsids to stall at packaging sites and that M53 mediates MCMV capsid localization to the nuclear periphery [15]. Subsequently, it was shown in an interesting paper [16] that an HSV-1 mutant, in which two basic patches of the N-terminal segment of its UL53 homolog, UL31, were made less basic, is defective for nuclear egress. IFA of UL31 and capsid proteins, particularly in cells transfected with a BAC containing the mutant genome, led to the conclusion that the N-terminal segment is required to direct migration of nucleocapsids to sites of primary envelopment at the nuclear periphery [16].

While our initial CLEM results, using cells into which BACs containing the null mutant genome were introduced, raised the possibility that UL53 is important for capsid localization towards the nuclear periphery, subsequent analysis of a larger number of cells that were directly and synchronously infected by null mutant viruses indicated that deletion of *UL53* (or *UL50*) did not result in altered intranuclear localization of capsids. (We also observed that copious DNA-filled C capsids formed in the absence of UL53, as we had previously [10], indicating that UL53 is not essential for viral DNA packaging or for stabilization of C capsids.) It is possible that UL53 differs from its EBV, MCMV, and HSV-1 homologs in its ability to promote capsid migration to the nuclear rim. It is also possible that certain differences in results among the systems might reflect differences in how experiments were performed such as differences in preparation of infected cells (e.g., transfection vs. infection), mutants used (e.g., null vs. ones retaining certain activities), and whether and how intranuclear distributions of capsids were quantified.

What role does UL53 interactions with nucleoplasmic capsids play? One possibility is that these interactions are transient and/or infrequent and play no role. A second speculative possibility is that any UL53 that associates with capsids at the RC and migrates with them to the nuclear rim is then able to dock those capsids to any unoccupied UL50 at the INM for subsequent primary envelopment. Such a role would not depend on whether capsids migrate by the nuclear actomyosin machinery [5] or by random diffusion [31,32]. Additional studies are required to address these possibilities.

## Data Availability

Data supporting the reported results can be found in the figures and the Supplementary Materials.

## Supplementary Materials

The following are available online at https://www.mdpi.com/article/10.3390/v14030479/s1, Table S1: Viral proteins detected in FLAG-immunoprecipitates from nuclear lysates of cells infected with 53-F, but not wild-type HCMV; Figure S1: CLEM analysis of intranuclear distribution of capsids.
