# Supplementary material for: Human Cytomegalovirus Nuclear Egress Complex Subunit, UL53, Associates with Capsids and Myosin Va, but Is Not Important for Capsid Localization towards the Nuclear Periphery
Source: Viruses. 2022 Feb 26;14(3):479. doi: 10.3390/v14030479 (PMC8949324; doi:10.3390/v14030479)
Supplement: Supplementary file 1 [file viruses-14-00479-s001.zip › viruses-1594599-supplementary.pdf]

Table S1
Viral Proteins Detected in FLAG-Immunoprecipitates from Nuclear Lysates of Cells Infected with 53-F, but not Wild-Type HCMV

Molecular weight band	# of peptides	Viral protein	Viral protein function	Coverage (% of total amino acids)
150-250kD	6	MCP	Major capsid protein	6
100-150kD	19	MCP	Major capsid protein	19
	9	TRS1	Capsid assembly	9
	7	pp150	Tegument protein	7
	7	UL50	NEC subunit	7
	7	UL56	Terminase subunit	7
	6	UL89	Terminase subunit	6
75-100kD	15	UL104	Capsid portal protein	21.9
	11	UL89	Terminase subunit	15.4
	11	UL25	Tegument protein	20.1
	10	UL50	NEC subunit	24.9
	5	US23	Tegument protein	7.9
	4	TRS1	Capsid assembly	4.7
	3	pp65	Tegument protein	6.1
	2	UL97	Ser/Thr kinase	4.4
50-75kD	8	pp65	Tegument protein	24.3
	2	UL50	NEC subunit	6.2
30-50kD	17	UL53	NEC subunit	31.9
	17	UL50	NEC subunit	39.8
	8	UL44	Processivity factor	19.9
	17	UL53	NEC subunit	31.9
	17	UL50	NEC subunit	39.8

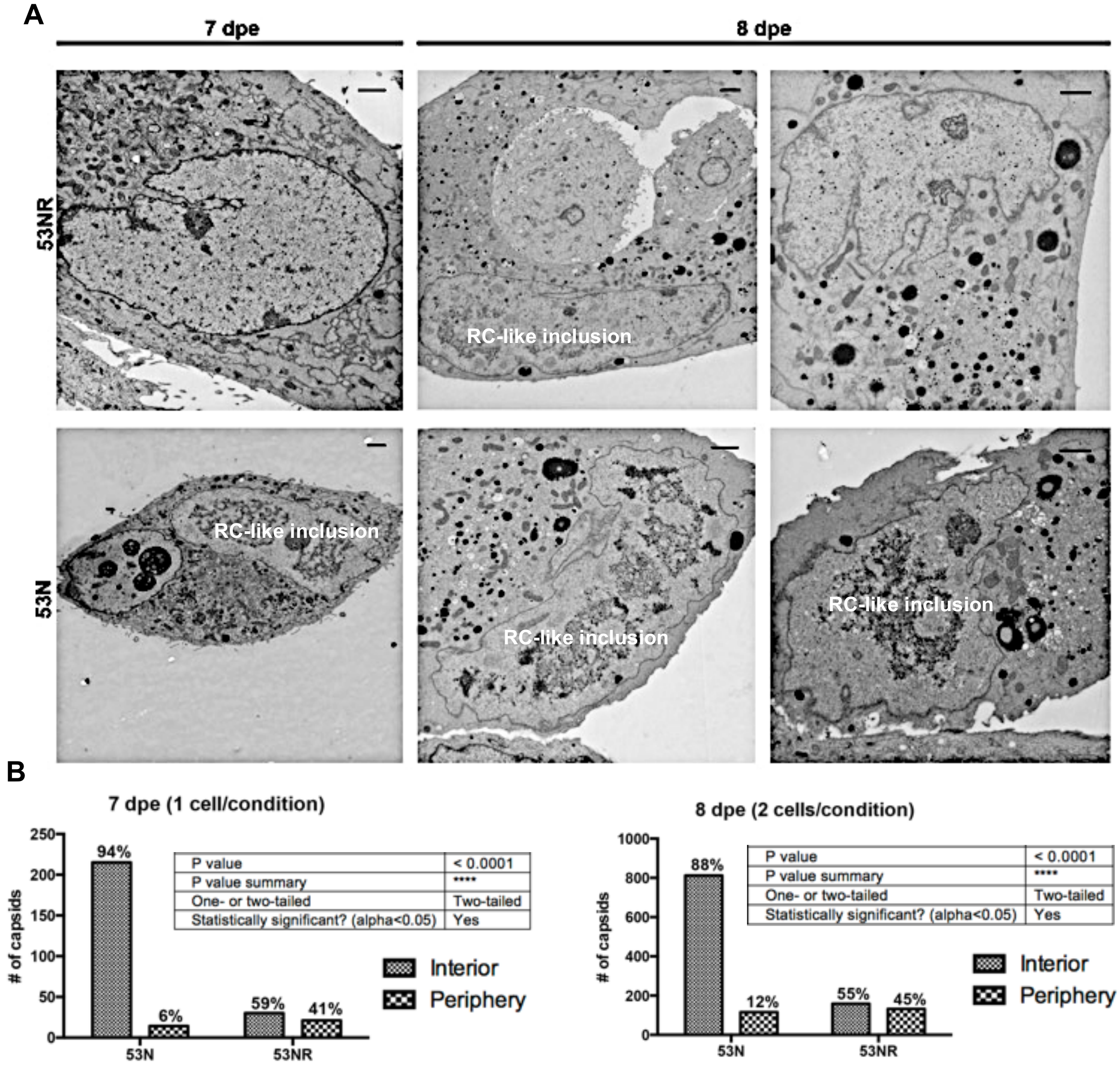

Figure S1: CLEM analysis of intranuclear distribution of capsids. HFFs were electroporated with 53N or 53NR HCMV BAC DNA, fixed at 7 or 8 dpe, and processed for CLEM. The percentage of capsids located within 2μm of the nuclear rim was calculated (scale bars are 2μm). (A) Shows micrographs of whole cell sections of each cell analyzed. The pair of cells from 7 dpe are also shown in Fig 2 from [10]. (B) Shows capsid counts and statistical calculations using Fisher's exact test.
